# Dental Histology

**Published:** 1876-09

**Authors:** W. H. Atkinson


### DENTAL HISTOLOGY.

BY DR. W. H. ATKINSON.

*Remarks before the Brooklyn Dental Society.*

The word histology is derived from the Greek words, *histos*, a web, and *logos*, a discourse, and means a discourse about webs. Web spinning and web weaving involve the prepation of the elements of tissues and the putting them in place to form organs. This history of this process is true histology. A full knowledge of the laws and forces governing and displayed in the upbuilding of tissues, and their aggregations would enable the observer to delineate the mode of production and function, special and general, of any specimen brought to him for examination. A wonderful display of this regnancy of law is to be witnessed in cocoons; these discover to the educated mind the generic and specific characters of the weavers of these wondrous webs. Dumb bell cocoons, cylindrical cocoons, oval and ovoid cocoons, are constantly the proper products of the varieties of workers to which they respectively belong, so that the silk culturalist is enabled to select his stock with certainity by sight of the cocoons. Experts are said to be able to define not only species and varity, but also sex, of the cocoons, upon special inspection.

It will be apparent that observation of the career of the silk worm was the means of learning this lesson; but we have no such obvious example in the career of tooth elements to enable us to discover so exactly the inception, career and disappearance of the elements and bodies that construct the teeth. Not until within a very late period was there any just apprehension of the existence or character of the tissue weavers of the tooth elements—the cells—that construct the pulp, dentine, enamel, and cement, which are properly dental tissues, and are the legitimate bodies for our consideration on this occasion. The microscope has made it possible for us to see these minute bodies that have been denominated cells.

It also reveals to sight the parts of these minute structures in clearest detail, as body, wall, nucleus, nucleolus, granule, etc., by which we are able to differentiate one portion of a cell from another, and also to see the difference between the cells that produce the dentine, enamel and cement, and also to learn their relations in normal aud abnormal presentment. To the microscope, then, we must appeal for the settlement of all questions that arise as to health or disease in these minute bodies and structures. Here, as in the other departments of minute (and coarse) anatomy, we must rely more upon examinations of the actual specimens than upon the writings or opinions of other observers, if we wish to be at all certain of our principles or the practice based thereon. This will suggest the high propriety of this society taking up and thoroughly investigating the subject of "Dental Histology." If we are unable to settle a single dispute, the turning of our minds in this direction will at least cause us to learn what we do not already know in some degree, and also help to clear the way for us to propound intelligible interrogatories to each other, and thus stimulate investigation and personal examination into the already broken ground for research.

Dental histology springs so many questions upon us that it seems a great undertaking to consider them; even the least of which forces us to consider the origin of each body and process set before us.

This present effort of mine was sprung by the President putting my name down as one of the speakers upon the subject of the evening. Whatever may come of our endeavor is attributable at least to this appointment as an exciting cause. Will it not be profitable for us to inquire whether the demand for some special exercise of our powers such as this be not a piece with each and every performance of body and process throughout the whole domain of work and worker? All known bodies originate from elemental constituents so unlike the perfected body as to utterly preclude us from knowing the adult by an examination of the germ, or the germ from an adult form of body before us. If, then, we can only deduce function by observing bodies at work, and know of no body but has been called into being by the demand of work needed to be done, is it not clear that function is before body and the limit of its plan and power to do that for which it was destined?

The builders of tissues are called cells; the elements of cells, granules; the elements of granules, molecules, and the elements of molecules, atoms. This carries us back as far as a mere physical survey is capable of conducting us; and we are as helpless here as at any stage of the progress, from a complete tooth down the ladder of construction to this the starting part of all an-atom-y of all possibility of physical body. If, then, we would know the true history (histology) of the final elements of dental tissues, it is manifest that we must give attention to these profound studies, and become by familiarity there with, truly erudite histologists of "dental tissues."

What does all this involve? To begin: Chemistry is demanded, which, when fully apprehended, suggests and prophesies all the rest of natural history, including biology and psychology. Chemistry supplies us with the materials; biology gives them form and arrangement; psychology enables us to apprehend and comprehend them in becoming factors of apparent functions other than those of atomic or chemical form.

Elements of tissues may be divided into classes, *i. e.*, simple and compound, unitary and mutiple, ultimate or obsolute, and proximate.

Coherence and adherence owe their presence to the affinities that produce them.

A display of degrees of affinities has been observed in what has been called "saturation," or satisfaction of affinities, and "non-saturation," or partial satisfaction of affinities. The bodies produced by these diverse methods, are diverse in many characters; the principle of which is seen in the stability of the bodies resulting from saturation, and the instability of those resulting from non-saturation of the producing currents. The bodies of the mineral kingdom are the most stable bodies of which we have any knowledge; those of the vegetable kingdom are less stable, and those of the animal kingdom least stable of all. The length of the stay of elements together without modification, is a key to the grade of body they form—that is, fully saturated bodies of the binary group are the longest lived, and the lowest in endowment of presence and power. These have no outside affinities until their elements are dissociated by violence from without. The next group in order is displayed in non-saturated bodies—that is to say: two elements in union, one element of which has one bond or power of affinity, and the other possessing two measures or bonds of affinity, only one of which is engaged to saturate that of number one, thus leaving one bond still free to seek or accept its saturating affinity whenever presented. This last is exemplified in the affinities of hydrogen and oxygen, when forming water, H—O—H, hydrogen oxide: or H—O—O—H, hydrogen or peroxide, or oxygenated water. There are sixty-three kinds of atoms now known, thirteen of which conspire to form the human body; six enter into the structure of tooth tissue proper, viz., oxygen, hydrogen, carbon, nitrogen, lime and phosphorus.

Atoms can not be divided, but they can be aggregated; and the methods of aggregation present us with elements and proximate elements, with which we must deal if we wish to understand the constructions and destructions of the tissues, with which we are now called upon to interest ourselves. We must possess in some degree this knowledge, before we can hopefully undertake to arrest or restore the destructive metamorphosis that constitutes that which we call decay.

All metamorphosis of tissue is decay proper of some form of body, and therefore not to be deprecated in this sense. Premature decay is all that the most fastidious student of a pure physiology could desire to prevent, arrest or restore. The object of our present efforts to stimulate to the study of "Dental Histology" can only be gained by observing the influence of the sun upon disintegrated mineral substance, combining and rising into secretory or cell tissue, which, under the continuance of the same influx, produces the various forms of vegetable growths, from the merest spore and fungus forms, that die of old age by the million in an hour, to the most enduring giant of the forest that flourishes for thousands of years, and then only dies by accident, and not by the exhaustion of the production of the typical cells, whose duty it is to ever stand as the high priest, to marry the saturable bonds of the cells to each other, producing mineral again (of modified form), on the one hand, and the non-saturated bonds to the pabulum, in sap, to form new secretory cells to perpetuate the cambium between bark and wood on the other,

Certain rhythmical undulations, or movements of sun influence, are concerned in all the synthetical combinings of simple elements into proximate elements; and these into tissues, organs and systems.

Any condition that disturbs the rhythm of these movements of sun presence, arrests, modifies or reverses the order of combination of elements. In case of arrest, the composition is prevented for a time, to be resumed again without material deteriment to the ultimate product. In cases of modification imperfect products will follow, in degrees of poverty of endowment according with the modificatory force. In cases of reversal of sun presence, we are presented with disintegratory motions, in the inverse order of the rymthmical synthesis.

The great generalization of the principles and affections of the elements of tissues that an observation brings to plainest apprehension, is, that association, once enjoyed among the constituents, absolute or proximate, is ever dear to, and sought to be maintained by, them. We have already instanced the permanence of the associations of binary elements and the whole range of saturated constituencies of bodies. Thus do we trace the primal impulse of nature from the almost immutable enamel up through more mutable, because more complicated, forms of matter, solid, liquid, gaseous and protonaceous, till it reaches the summit of the scale in the human brain, producing there the divinest and most evanescent association known to the elements of tissues, as such; and thus we are learning the lesson of mutual dependence, support and love, that reconciles us to the cancellation of the puerile bonds once held so dear. And we gladly accept the new surprises of the influx of sun influences, that we are now convinced will safely conduct to the highest destiny! Am I not then justified in the assertion of the mutual dependence of all departments of being, of science, and of method of presentment of these, in the associative and dissociative activity in matter and mind that acquaint us therewith, and enable us to perceive the grand synthesis by the detailed analysis of presence and power, whose mutations constitute not only our bodies and minds, with their physical and psychical gyres, but our individual and social relations of affection to every atom, molecule, organ, system and cycle of systems, that constitute the blessed Father's house of many mansions, which we denominate the Universe?

Mr. President and gentlemen, I trust you now see we have before us no puerile or insignificant work, if we attempt to understand the creation or *becoming of tissues*, and their aggregations into organs and systems. A complete apprehension of the production and use of a human tooth involves, in its degree, the laws and processes by which the universe itself is produced and maintained; and although all the past stages of apprehension and scientific attainment are necessary and indispensable, yet I wish to warn you against accepting them as ultimate truth; for all the processes of biology and psychology involve chemistry and physics, natural and (what is called) voluntary modes of association and dissociation of primates in physics and consciousness, that are themselves progressive, as we become able to apprehend them, by increased facilities of discovery and invention, by which the processes of understanding are shortened and rectified to such a degree as to necessitate the discarding or rewriting of text books. So painfully apparent is this fact, that we may well be discouraged if we expect to be fully apprehended and understood in such efforts as these, to compress into short and aphoristic statements the scientific processes, natural processes and functional processes of the bodies with which we have to deal.

Sept-3

The teacher is constantly under the necessity of making many statements or parts of statements that must remain for future comprehension in demonstrative degree, hoping for an understanding of a part only of that which is attempted to be demonstrated. This arises more from the nature of things in their progressive necessities, and the fallacy of former teachings than from our inability to understand alike the complexity and simplicity of the analysis and synthesis of bodies and processes that appear as simple to the unaided sight, but which are discovered to be composite the moment the factors of function are revealed. The plan of the universe must have preceded its production. No naturalist can be an atheist, for he is ever in the presence of potential or actual functions, that always operate by specific modes, denominated laws.

Now, I trust you see whether or not we have any thing to waken our enthusiasm, to engage the utmost mental tension, and absorb our affections, thus making the study of dentistry dear to our hearts. No one desires to be deceived for he feels that he is entitled by birth right to the truth, and to come to the full fruition of typical endowment in body, mind and efficiency. So soon as this spirit shall imbue our hearts, mere money getting will lose its charm, and the desire to conquer the problems before us will so possess our affections as to make the interest to know the certainity of the propositions we investigate a common passion of a common brotherhood seeking the truth. Then there will be no contention among us, but that noble emulation of who can best work, and who can best agree.

Now, since cells have been revealed as the factors of function, we are delivered from a host of misapprehensions that formerly obstructed the way. That type holds undisputed dominion throughout the universe, is made evident to us by the cancellations of the elements of tissues and organs in alternation, and the retention of functionless organs, as instanced in the embryonal shedding of teeth in the Cetacea and Marsupialia, and the functionless mammary glands in male animals and man. As has been already stated, function precedes factor of function or organ; use, the thing that operates the use. The ideal man requires the preparation of his food; this ideal or typical necessity potentially evokes the organ of mastication, that when produced upon this plan comminutes the food. The method by which plan is wrought out in the human body is tolerably clearly understood, and very fairly detailed, in the late works on "Dental Histology," and may be succinctly stated as follows: The first apparent change is called the granular stage, and constitutes, as its name implies, an accumulation of granules, that in process of time becomes the secondary stage, called the papillary, which merges into the third stage, denominated the follicular.

When the papules that constitute the sides of the follicle have increased sufficiently to unite and close the mouth of the follicle by fusing together, we have the saccular stage, inclosing the germ of the tooth, called the tooth pulp, consisting of two planes of cells, destined to calcification, according to the law of their production, as demanded by the use to which they are assigned. The enamel cells calcify immediately against the first stratum of dentinal cells, and a process of hardening proceeds in the enamel cell, toward the periphery of the pulp, until complete. The dentinal cells calcify toward the center of the pulp until the series of cells are exhausted by calcification, and then we say the tooth is fully developed, leaving now the pulp in the pulp-chamber. The cementum is produced in like manner by characteristic cells, calcifying from the zonal or interglobular space toward the periphery.—*Dental Cosmos.*

